# Validity and measurement invariance of the *Screening Instrument for Students At-Risk of Dropping Out* in health sciences students

**DOI:** 10.3389/fmed.2026.1941240

**Published:** 2026-09-18

**Authors:** Carlos Alberto Henao Periañez, Cruz Deicy Jaramillo-Bolívar, Marcio Alexander Castillo-Díaz

**Affiliations:** 1 Escuela de Enfermería, Facultad de Salud, Universidad del Valle, Cali, Colombia; 2 Programa de Enfermería, Facultad de Ciencias de la Salud, Universidad Libre, Cali, Colombia; 3 Departamento de Psicología/Maestría en Psicometría y Evaluación Educativa, Facultad de Ciencias Sociales, Universidad Nacional Autónoma de Honduras, Tegucigalpa, Honduras

**Keywords:** academic burnout, dropout intention, health sciences education, measurement invariance, structural validity, student satisfaction, undergraduate students

## Abstract

**Background:**

University dropout remains a major challenge in higher education, particularly in health sciences programs, where academic demands are high and retention is critical for workforce sustainability. Psychometrically sound instruments for assessing constructs associated with the dropout process remain limited, especially in Latin American contexts. This study aimed to evaluate the psychometric properties and measurement invariance of a *Screening Instrument for Students At-Risk of Dropping Out* among health sciences students from public and private universities.

**Methods:**

A cross-sectional methodological study was conducted using secondary data from two universities in Cali, Colombia (public: *n* = 320; private: *n* = 437; total *n* = 757). Confirmatory factor analysis (CFA) was performed to assess the three-factor structure of the instrument (satisfaction with education, academic burnout, and dropout intention). Internal consistency was evaluated using McDonald's omega (*ω*) and ordinal alpha (*α*). Convergent and discriminant validity were assessed through average variance extracted (AVE) and heterotrait-monotrait ratio (HTMT), respectively. Measurement invariance (configural, metric, and scalar) was examined across institutional context, gender, and working status. Latent mean differences were subsequently estimated.

**Results:**

The three-factor model showed an acceptable fit (CFI = 0.964, TLI = 0.954, SRMR = 0.057, RMSEA = 0.080). Factor loadings were significant and substantial (*λ* = 0.706–0.964). Internal consistency was high across subscales (*ω* = 0.863–0.978; *α* = 0.860–0.978). AVE values exceeded 0.50, and HTMT values were below 0.85, supporting convergent and discriminant validity. Measurement invariance was supported across all grouping variables (*Δ*CFI ≤ 0.006; *Δ*RMSEA ≤ 0.005). Latent mean comparisons indicated that students from the participating public university reported higher levels of satisfaction with education, academic burnout, and dropout intention than those from the participating private university. Female students showed lower dropout intention than males.

**Conclusions:**

The instrument demonstrated adequate psychometric properties and measurement invariance across key groups, supporting its use for comparative analyses. These findings support its use for assessing and comparing satisfaction with education, academic burnout, and dropout intention among health sciences students. However, these constructs, particularly dropout intention, should not be interpreted as equivalent to actual dropout risk. Further longitudinal research is needed to determine its ability to predict actual dropout and support individual-level screening decisions.

## 1 Introduction

University dropout remains a persistent issue in higher education, with significant implications for institutional efficiency, educational equity, and the development of human capital (1). In health sciences programs, this phenomenon is particularly critical due to the high academic demands, the costs associated with professional training, and the need to ensure the sustainability of healthcare systems (2). Health sciences education also involves intensive workloads, demanding schedules, clinical training, and exposure to patient care, which may increase emotional strain and academic burnout and influence students' satisfaction and intentions to persist in their studies (3–5). In this context, dropout not only affects individual trajectories but also compromises the future availability of professionals in critical areas (6).

University dropout should not be understood as an isolated event, but rather as a dynamic and cumulative process that develops over time through the interaction of multiple individual, academic, and contextual factors (7, 8). Classical models, such as those proposed by Vincent Tinto (9) and John P. Bean and Barbara Metzner (10), conceptualize dropout as the result of insufficient academic and social integration, as well as external pressures that affect student persistence. More recent evidence highlights the central role of psychological variables—particularly academic burnout, satisfaction with education, and dropout intention—as proximal predictors of student attrition (11, 12).

Dropout intention refers to a student's conscious and self-reported inclination or plan to leave higher education before completing their degree (12). In particular, it has been shown to be a robust predictor of actual dropout, reinforcing its relevance as a key construct for early identification (8). This construct involves thoughts about the possibility of changing or leaving higher education (10, 13), and not only encapsulates the risk of attrition but also enables the identification of vulnerability trajectories before dropout materializes (6, 14).

Recent studies among health sciences students have shown that academic burnout constitutes one of the main predictors of dropout intention, whereas satisfaction with education acts as a protective factor, moderating the impact of academic demands on student persistence (15). This integrative approach, which simultaneously considers risk and protective factors, has been highlighted as essential for understanding university dropout in complex contexts (10).

Given the complexity of the phenomenon, there is growing interest in the use of screening instruments to identify students at risk of dropout at early stages. These tools are essential for informing timely interventions and institutional decision-making (16). However, the availability of psychometrically robust instruments remains limited, particularly in specific populations such as health sciences students. Existing measures often focus on isolated dimensions (e.g., satisfaction or engagement) rather than capturing the multidimensional nature of dropout risk (17–19).

In this context, the *Screening Instrument for Students At-Risk of Dropping Out* represents a relevant approach, as it integrates multiple dimensions of dropout risk within a single instrument (20). However, the available evidence regarding its psychometric properties remains limited, being largely restricted to the original validation study and applications in specific contexts, predominantly among Portuguese-speaking populations (21–23). Factorial structure analysis provides key information on the latent dimensions underlying the items and their overall model fit, which is essential for establishing the structural validity of the instrument in new contexts (24). This limitation constrains the generalizability of its findings and underscores the need to validate the instrument across diverse cultural and educational settings.

In Latin America, validation studies of instruments aimed at identifying the risk of university dropout remain limited and are particularly scarce in specific populations such as health sciences students. Recent evidence from Colombia has shown that dropout intention is associated with academic burnout, satisfaction with education, and other personal, academic, and psychological factors among university and health sciences students (15, 25). However, these studies have focused on the relationships between dropout intention and its associated factors rather than on the measurement properties of the instrument. The lack of psychometric studies in Latin American contexts hinders the understanding of the cultural nuances that latent variables may exhibit in these settings. Therefore, evidence regarding the structural validity and measurement invariance of the *Screening Instrument for Students At-Risk of Dropping Out* remains limited in Colombian health sciences students. This limitation is especially relevant given that the psychometric properties of instruments may vary according to language, cultural context, and sample characteristics, making it necessary to conduct context-specific evaluations to confirm construct validity, internal consistency, and the practical utility of these tools (26).

From a psychometric perspective, instrument validation requires rigorous evaluation of its internal structure, as well as the assessment of its equivalent functioning across different groups (27). Measurement invariance analysis in these contexts allows determining whether the instrument operates equivalently in populations exposed to different structural conditions (28), thereby strengthening its applicability in comparative studies and cross-cultural contexts in Latin America. In the absence of such evidence, it is not possible to ensure that observed differences between groups reflect true variations in the constructs being assessed, as they may instead result from differential functioning of the instrument across groups.

In Colombia, public and private universities operate within distinct institutional contexts, particularly regarding funding mechanisms, tuition costs, access, and the socioeconomic composition of their student populations (29). Public higher education institutions receive substantial government funding and are currently supported by a national tuition-free policy for eligible undergraduate students (30). These institutional differences may shape students' educational experiences and provide a relevant rationale for examining whether the instrument functions equivalently across public and private university contexts before comparing latent construct levels between them.

In this context, the present study aims to evaluate the psychometric properties of the *Screening Instrument for Students At-Risk of Dropping Out* among health sciences students by examining its structural validity, internal consistency, and measurement invariance across institutional context (public vs. private universities), gender, and working status. The validation of this tool in this population is essential to ensure its appropriateness, precision, and utility as a screening instrument in health education programs.

To the best of our knowledge, this study represents one of the first psychometric evaluations of this instrument in a Latin American context different from that of its original development, thereby contributing to addressing a critical gap in the measurement of dropout risk in higher education. Additionally, the evidence generated by this study may inform future early identification strategies and institutional initiatives aimed at promoting student retention within a comprehensive academic well-being framework.

## 2 Method

### 2.1 Study design

A methodological study was conducted to evaluate the psychometric properties of the instrument using a cross-sectional design. The analysis was based on secondary data derived from two independent studies carried out in higher education settings. The study followed the recommendations of the COSMIN initiative (Consensus-based Standards for the Selection of Health Measurement Instruments) for the evaluation of validity and reliability of measurement instruments (27).

### 2.2 Setting

The data were obtained from two universities located in Cali, Colombia: one public and one private institution, both with faculties of health sciences. These institutions offer undergraduate programs in health-related disciplines and share comparable academic structures, although they differ in institutional characteristics and student profiles. This context allowed for the evaluation of the instrument across distinct educational environments within the same geographical setting.

### 2.3 Participants

In the public university, the target population consisted of 1,503 undergraduate students distributed across seven programs: Medicine (*n* = 318), Nursing (*n* = 197), Physiotherapy (*n* = 185), Bacteriology (*n* = 175), Speech and Language Therapy (*n* = 173), Occupational Therapy (*n* = 234), and Dentistry (*n* = 221). In the private university, the target population comprised 1,394 undergraduate students enrolled in health sciences programs, including Medicine (*n* = 840), Nursing (*n* = 373), and Psychology (*n* = 181).

The required sample size was estimated using an *a priori* power analysis for structural equation modeling. Assuming a small-to-moderate anticipated effect size (0.20), a statistical power of 0.80, and a significance level of 0.05, the specified model—including three latent variables and 12 observed indicators—yielded a recommended minimum sample size of 296 participants per institution to ensure adequate statistical power and model stability (31).

In both universities, academic programs were used as strata, and the number of students selected from each program was determined proportionally to its size within the corresponding eligible population. Within each stratum, eligible students were identified from institutional enrollment lists and selected by simple random sampling using computer-generated random numbers. Selected students were contacted through institutional communication channels and provided access to the online questionnaire. When necessary, additional students were randomly selected using the same procedure to compensate for non-response while maintaining proportional representation across academic programs. Complete recruitment-flow information was not prospectively retained in both original studies; therefore, the cumulative number invited and the number who accessed or started but did not submit the questionnaire could not be reliably reconstructed for this secondary analysis. The final analytical sample comprised 757 students with complete responses to the items evaluated in the present study: 320 from the public university and 437 from the private university.

### 2.4 Inclusion and exclusion criteria

Participants were eligible for inclusion if they were 18 years of age or older, were actively enrolled in undergraduate programs within the Faculty of Health during the 2025–1 and 2025–2 academic terms, and provided voluntary informed consent to participate in the study. Students were excluded if they declined participation and did not provide informed consent.

### 2.5 Data collection instruments

Data were collected through a structured, self-administered questionnaire administered online using Google Forms. Survey links were distributed to selected students through institutional communication channels. The items included in the present psychometric analysis were configured as mandatory, and the “Limit to 1 response” option was enabled to prevent multiple submissions by the same participant. Consequently, the dataset used for this secondary analysis contained complete responses for all items evaluated. The instrument included sections on sociodemographic and academic characteristics, as well as measures of constructs related to risk of dropout.

#### 2.5.1 Sociodemographic and academic variables

Sociodemographic and academic information was collected using a questionnaire designed for this study. The variables included age (in years), gender (female, male, or other), institutional affiliation (public or private university), academic program, and time at university (categorized as less than one year or one year or more). Additional variables included place of origin (Cali vs. Outside Cali), socioeconomic status (based on the Colombian classification system, which considers residential location and access to public services), and employment status (working while studying vs. not working). Academic performance was assessed through self-reported grade point average (GPA), categorized according to institutional grading ranges. These variables were selected based on their relevance in prior research on academic persistence and dropout.

#### 2.5.2 Assessment of risk of dropout-related constructs

The *Screening Instrument for Students At-Risk of Dropping Out* (20) was used as a self-report measure grounded in a multidimensional theoretical framework of university dropout. The instrument consists of three subscales—academic burnout, satisfaction with education, and dropout intention—each composed of four items. Responses are recorded on a five-point Likert scale ranging from 1 (strongly disagree) to 5 (strongly agree), with higher scores indicating higher levels of the respective construct.

Previous studies have provided evidence supporting the validity and reliability of this instrument among university students, including adequate internal consistency across its subscales: academic burnout (*α* = 0.83; *ω* = 0.83), satisfaction with education (*α* = 0.81; *ω* = 0.80), and dropout intention (*α* = 0.76; *ω* = 0.75) (20).

A Spanish version of the *Screening Instrument for Students At-Risk of Dropping Out* was used in this study. Prior to its administration, the instrument underwent a qualitative review by three expert judges with complementary backgrounds—a psychologist, a nurse, and a pedagogue—to ensure its suitability for the target population. The review assessed four types of equivalence: semantic (accuracy of meaning), idiomatic (appropriateness of language use), experiential (relevance to students' academic context), and conceptual (consistency with the underlying constructs). The original English wording of each item, its corresponding Spanish-language version, and the theoretical dimension to which it belongs are presented in Supplementary Table S1.

Subsequently, a pilot test was conducted with 30 students from the target population to assess the feasibility of the data-collection procedure and the clarity and comprehensibility of the questionnaire, including the version of the Screening Instrument used in this study. The pilot test did not involve formal cognitive interviews or quantitative ratings, as its purpose was not to provide additional psychometric evidence. Students were able to understand and complete the questionnaire as intended, and no difficulties requiring modifications to the questionnaire or data-collection procedure were identified.

### 2.6 Ethical considerations

The study was conducted in accordance with national and institutional ethical standards for research involving human participants. Ethical approval was obtained from the Research Ethics Committee of Universidad del Valle, Cali, Colombia (Record No. 093-024, November 6, 2024), and from the Research Ethics and Bioethics Committee of Universidad Libre, Cali Campus (Act No. CAL-131202409-CE, November 8, 2024). The study adhered to the principles established in Resolution 008430 of 1993 issued by the Colombian Ministry of Health.

Prior to participation, all students received detailed information regarding the objectives and procedures of the study and provided voluntary informed consent. Participation was anonymous and confidential, and students were informed of their right to withdraw at any time without academic or other consequences. The study was classified as minimal risk, and no ethical concerns or adverse events were reported during the data collection process.

### 2.7 Data analysis

Statistical analyses were performed using the RStudio environment (version 2025.5.1.513) with R (version 4.4.2) (32). Confirmatory factor analyses were conducted using the *lavaan* package (version 0.6–16) (33), complemented by the *semTools* package (version 0.5–6) (34).

Descriptive statistics were first calculated to characterize the study sample. Continuous variables were summarized using means and standard deviations, whereas categorical variables were described using frequencies and percentages. Item-level descriptive analyses were subsequently conducted for the *Screening Instrument for Students At-Risk of Dropping Out*, including means, standard deviations, skewness, and kurtosis. Skewness and kurtosis were examined descriptively to characterize item-response distributions and were not used as criteria for assessing or establishing univariate normality.

The dimensional structure of the *Screening Instrument for Students At-Risk of Dropping Out* was examined through item-level confirmatory factor analysis (CFA), specifying the original three-factor model corresponding to the theoretical structure of the instrument: satisfaction with education, dropout intention, and academic burnout (20). The model was estimated using the weighted least squares mean and variance adjusted (WLSMV) estimator, which is appropriate for ordinal data and provides robust standard errors and a mean- and variance-adjusted test statistic. The reported *χ*^2^, CFI, TLI, and RMSEA correspond to the robust fit statistics provided by *lavaan* under WLSMV estimation (24). Alternative factorial models were not evaluated because the original instrument was theoretically defined as comprising three distinct dimensions, and no alternative factorial structure has been theoretically proposed for the instrument. Therefore, the CFA focused on testing the originally hypothesized three-factor structure.

Model fit was evaluated using multiple goodness-of-fit indices, including the Comparative Fit Index (CFI), Tucker–Lewis Index (TLI), Root Mean Square Error of Approximation (RMSEA), and Standardized Root Mean Square Residual (SRMR). Following commonly accepted criteria, model fit was considered adequate when CFI and TLI values were ≥0.95 and RMSEA and SRMR values were ≤0.08 (35).

Standardized factor loadings (*λ*) were inspected to assess the contribution of each item to its corresponding latent factor, with values ≥0.40 considered acceptable. Internal consistency was evaluated using McDonald's omega (*ω*) and ordinal alpha (*α*), with values ≥0.70 interpreted as indicative of adequate reliability (36, 37). Convergent validity was examined using the average variance extracted (AVE), and discriminant validity among latent variables was assessed using the heterotrait-monotrait ratio of correlations (HTMT). HTMT estimates were calculated using polychoric correlations among the ordinal items, with values <0.85 considered evidence of adequate discriminant validity (38, 39).

Measurement invariance was assessed across institutional context (public vs. private university), gender, and working status using multigroup CFA for ordinal indicators. All 12 items were specified as ordered categorical variables, and models were estimated using WLSMV with the theta parameterization. Following established recommendations for ordinal measurement invariance (40), a sequence of nested models was evaluated. The configural model specified the same three-factor structure across groups. The metric model additionally constrained the corresponding factor loadings to equality across groups, and the scalar model further constrained the corresponding item thresholds to equality across groups. Because the indicators were ordinal, scalar invariance was evaluated through equality of item thresholds rather than intercepts. Considering the known sensitivity of *χ*²-based comparisons to sample size and the specific requirements for nested-model comparisons under WLSMV estimation, measurement-invariance decisions were based on changes in robust approximate fit indices (41). Invariance was considered supported when changes in model fit between nested models met the following criteria: *Δ*CFI < 0.010 and *Δ*RMSEA ≤0.015 (42).

Once scalar invariance was established, latent mean differences between groups were examined. In these analyses, one group was specified as the reference category (latent means fixed to zero), while the means of the comparison group were freely estimated. The resulting estimates represent unstandardized latent mean differences relative to the reference group; no additional standardization was applied (43).

## 3 Results

### 3.1 Sociodemographic and academic characteristics

The sample included 757 students (private university: *n* = 437; public university: *n* = 320), with a mean age of 21.64 years (SD = 3.30). Most participants were female (69.75%) and had been enrolled for at least one year (92.21%) (see Table 1). Differences between institutions were observed. Students from the public university were older and more frequently reported working while studying (*χ*^2^ = 26.70, *p* < .001), whereas those from the private university more often reported middle/high socioeconomic (*χ*^2^ = 22.40, *p* < .001), status and being born in Cali (*χ*^2^ = 7.80, *p* = .005). Additionally, higher GPA levels (4.0–5.0) were more prevalent among students from the public university (*χ*^2^ = 98.30, *p* < .001).

**Table 1:** Sociodemographic and academic characteristics of health sciences students by university.

Variable	Total Sample (*N* = 757)	Private University (*n* = 437)	Public University (*n* = 320)
Age (years), Mean (SD)	21.64 (3.30)	21.06 (3.04)	22.46 (3.47)
Gender			
Female	528 (69.75)	307 (70.25)	221 (69.06)
Male	229 (30.25)	130 (29.75)	99 (30.94)
Place of birth, *n* (%)			
Cali	480 (63.41)	295 (67.51)	185 (57.81)
Outside Cali	277 (36.59)	142 (32.49)	135 (42.19)
Socioeconomic status, *n* (%)*			
Low	267 (35.27)	124 (28.38)	143 (44.69)
Middle/High	490 (64.73)	313 (71.62)	177 (55.31)
Time at university, *n* (%)			
<1 year	60 (7.92)	58 (13.27)	2 (0.63)
≥1 year	697 (92.08)	379 (86.73)	318 (99.37)
Grade point average (GPA), *n* (%)			
3.0–3.9	315 (41.61)	244 (55.84)	71 (22.19)
4.0–5.0	411 (54.29)	165 (37.76)	246 (76.88)
<3.0	6 (0.79)	5 (1.14)	1 (0.31)
Not yet available (first-semester students)	25 (3.30)	23 (5.26)	2 (0.63)
Working while studying, *n* (%)			
No	555 (73.31)	352 (80.55)	203 (63.44)
Yes	202 (26.69)	85 (19.45)	117 (36.56)

M, Mean; SD, Standard deviation; *n* (%), frequency and percentage; *, The Colombian residential stratification system is widely used as a proxy indicator of socioeconomic conditions; GPA, Grade point average (range: 0.0–5.0).

Participants were enrolled in a range of health sciences programs (see Supplementary Table S2). This distribution reflects the stratified sampling design, ensuring that the proportion of students included from each academic program was consistent with the structure of the target population in both universities. In the private university, students were primarily from medicine, nursing, and psychology, whereas in the public university, the sample was more evenly distributed across multiple programs, including medicine, occupational therapy, and dentistry.

### 3.2 Item-level descriptive statistics

Item-level descriptive statistics for the total sample and stratified by university are presented in Supplementary Table S3. Across the full sample, mean scores for satisfaction with education items ranged from 3.81 to 3.90. Dropout intention items showed mean values ranging from 1.78 to 1.96, whereas academic burnout item means ranged from 2.33 to 2.79.

When examined by institutional context, students from the public university reported consistently higher mean scores in satisfaction with education (range = 4.05–4.24) compared to those from the private university (range = 3.52–3.72). A similar pattern was observed for academic burnout, with slightly higher values in the public university group. In contrast, dropout intention scores were marginally higher among students from the public university compared to their private university counterparts.

Regarding distributional properties, skewness values were generally negative for satisfaction with education items, indicating a tendency toward higher response categories. In contrast, dropout intention items exhibited positive skewness, reflecting clustering at lower response levels. Academic burnout items showed mixed skewness patterns.

### 3.3 Validity based on internal structure

A confirmatory factor analysis supported the hypothesized three-factor model (satisfaction with education, academic burnout, and dropout intention), showing an overall acceptable fit to the data (CFI = 0.964, TLI = 0.954, SRMR = 0.057), although the RMSEA was slightly above recommended thresholds (RMSEA = 0.080, 90% CI [0.079–0.088]). Figure 1 presents the standardized solution of the confirmatory factor analysis.

**Figure 1:**
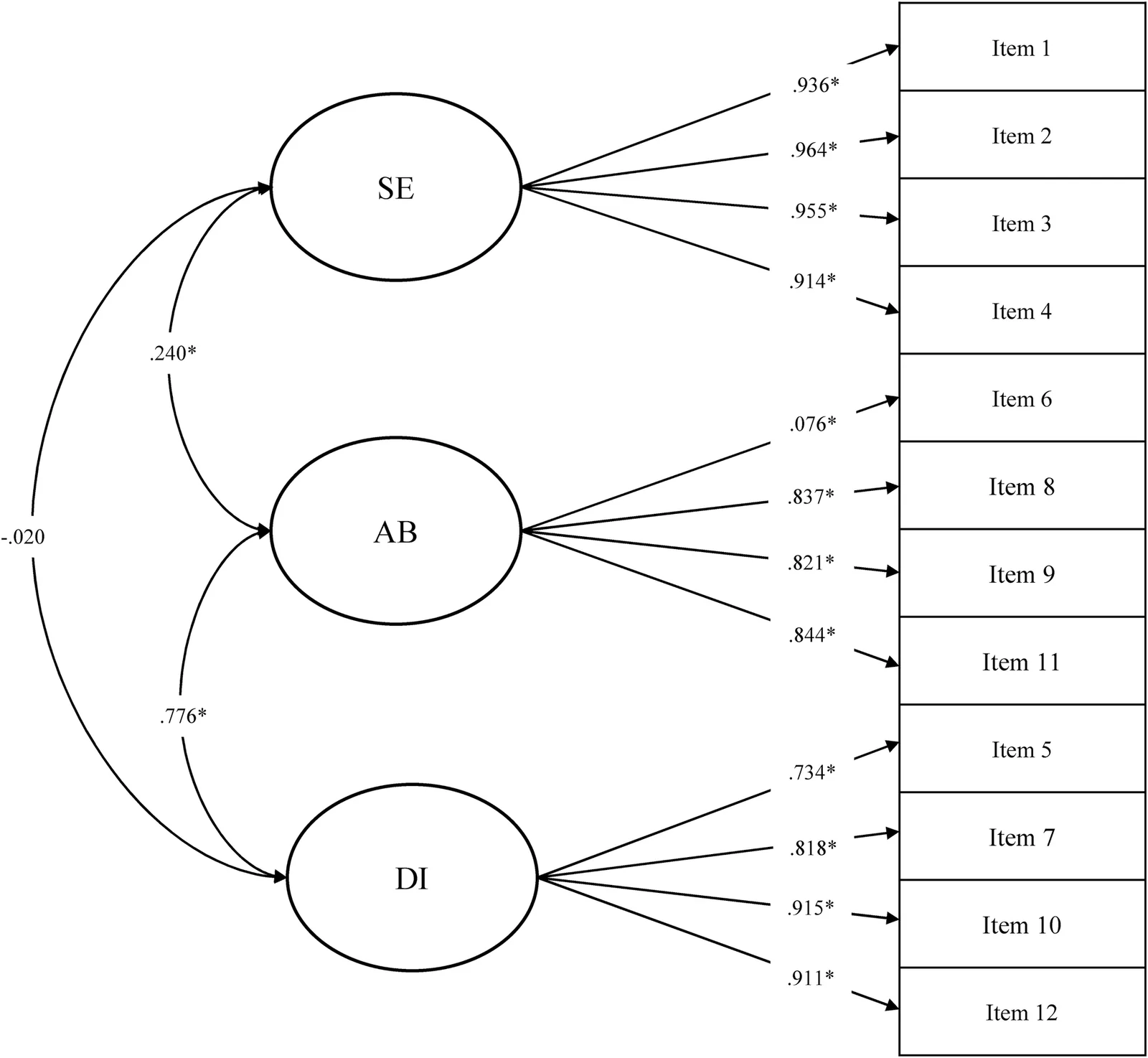
Confirmatory factor analysis model of the three-factor structure of the *Screening Instrument for Students At-Risk of Dropping Out*. SE, satisfaction with education; AB, academic burnout; DI, university dropout intention. * = *p* value < .001. Standardized factor loadings (*λ*) are presented for each item. Curved arrows represent correlations between latent factors.

All factor loadings were statistically significant (*p* < .001) and ranged from 0.706 to 0.964, indicating strong relationships between items and their respective latent constructs (see Supplementary Table S4). The highest loadings were observed for satisfaction with education, followed by dropout intention and academic burnout. Factor correlations showed a strong association between academic burnout and dropout intention (*r* = 0.776, *p* < .001), while satisfaction with education was weakly related to academic burnout (*r* = 0.240, *p* < .001) and not significantly associated with dropout intention (*r* = −0.020, *p* = .603). Overall, these findings support a multidimensional structure with three correlated factors.

### 3.4 Reliability, convergent and discriminant validity

The instrument demonstrated high internal consistency across the total sample and both institutional contexts. McDonald's omega and ordinal alpha coefficients exceeded recommended thresholds in all subscales (*ω* = 0.863–0.978; *α* = 0.860–0.978), indicating excellent reliability (see Table 2).

**Table 2:** Reliability, convergent and discriminant validity of the instrument across the total sample and by university.

Factor	Sample	*ω*	*α*	AVE
SE	Full sample	0.969	0.969	0.887
	Private university	0.978	0.978	0.917
	Public university	0.943	0.943	0.804
AB	Full sample	0.877	0.878	0.645
	Private university	0.863	0.860	0.609
	Public university	0.891	0.892	0.679
DI	Full sample	0.903	0.909	0.714
	Private university	0.895	0.903	0.696
	Public university	0.915	0.914	0.733

SE, satisfaction with education; AB, academic burnout; DI, dropout intention; α, ordinal Alpha; *ω*, McDonald's omega; AVE, Average variance extracted.

Evidence of convergent validity was supported by AVE values above the recommended threshold of 0.50 for all constructs across the total sample and both universities (range = 0.609–0.917). Satisfaction with education showed the highest AVE values, followed by dropout intention and academic burnout.

Discriminant validity was confirmed using HTMT ratios. All HTMT values were below the conservative threshold of 0.85 across constructs and groups, supporting adequate discriminant validity. The highest HTMT values were observed between academic burnout and dropout intention (0.732–0.774), indicating moderate but acceptable associations, while satisfaction with education showed comparatively lower associations with both constructs (see Table 3).

**Table 3:** HTMT ratios across groups.

Comparison	Full sample	Private	Public
SE—DI	0.027	0.058	0.121
SE—AB	0.246	0.356	0.050
DI—AB	0.756	0.732	0.774

SE, satisfaction with education; AB, academic burnout; DI, dropout intention; HTMT, Heterotrait–Monotrait Ratio.

### 3.5 Measurement invariance

Measurement invariance of the instrument was evaluated across three grouping variables—university, gender, and working status—with private university students, male students, and students who worked while studying serving as the reference groups. Across comparisons, the configural models showed adequate CFI values, although RMSEA values were elevated, indicating some degree of absolute model misfit (see Table 4).

**Table 4:** Measurement invariance of the screening instrument across groups.

Group	Invariance model	*χ*² (df)	CFI	*Δ*CFI	RMSEA (90% CI)	*Δ*RMSEA
University	Configural	440.21 (102)	0.960	–	0.094 (0.085–0.103)	–
	Metric	458.05 (111)	0.959	0.001	0.091 (0.082–0.100)	0.003
	Scalar	517.79 (120)	0.953	0.006	0.094 (0.085–0.102)	0.003
Gender	Configural	454.07 (102)	0.960	–	0.095 (0.087–0.105)	–
	Metric	456.35 (111)	0.960	0.000	0.091 (0.082–0.099)	0.004
	Scalar	473.98 (120)	0.959	0.001	0.088 (0.080–0.097)	0.003
Working status	Configural	426.19 (102)	0.963	–	0.092 (0.083–0.101)	–
	Metric	437.04 (111)	0.963	0.000	0.088 (0.079–0.097)	0.004
	Scalar	456.56 (120)	0.962	0.001	0.086 (0.078–0.095)	0.002

χ^2^, chi-square statistic; df, degrees of freedom; CFI, Comparative fit index; *Δ*CFI, change in comparative fit index; RMSEA, Root mean square error of approximation; CI, Confidence interval; *Δ*RMSEA, change in RMSEA.

Metric invariance was supported across all comparisons, as changes in fit indices were negligible (*Δ*CFI ≤0.001; *Δ*RMSEA ≤0.005), indicating that factor loadings are invariant across groups. Similarly, scalar invariance was achieved in all cases, with minimal changes in model fit (*Δ*CFI ≤0.006; *Δ*RMSEA ≤0.003). Overall, these findings provide evidence of measurement invariance, supporting the comparability of latent constructs across institutional context, gender, and working status.

Latent mean differences were examined across institutional and demographic variables using scalar invariant models (see Table 5). These estimates represent relative differences between each comparison group and its corresponding reference group and do not represent qualitative classifications of the constructs. Regarding institutional context, the participating public university showed significantly higher levels of satisfaction with education (latent mean difference = 0.462 [CI 95% = 0.295–0.629], *p* < .001), academic burnout (latent mean difference = 0.313 [CI 95% = 0.168–0.459], *p* < .001), and dropout intention (latent mean difference = 0.200 [CI 95% = 0.061–0.339], *p* = .005) compared with students from the participating private university. No significant gender differences were observed in satisfaction with education or academic burnout (*p* > .05). However, female students reported significantly lower levels of dropout intention than male students (latent mean difference = −0.192 [CI 95% = −0.346—−0.037], *p* = .015). Finally, no statistically significant differences were observed according to working status, suggesting that simultaneously studying and working was not associated with variations in satisfaction, burnout, or dropout intention (*p* > .05).

**Table 5:** Latent mean differences across groups.

Group	Factor	Latent Mean difference	95% CI	*p*
University	SE	0.462	0.295–0.629	<0.001
	AB	0.313	0.168–0.459	<0.001
	DI	0.200	0.061–0.339	0.005
Gender	SE	0.009	−0.183–0.200	0.927
	AB	−0.021	−0.179–0.137	0.796
	DI	−0.192	−0.346–−0.037	0.015
Working status	SE	−0.163	−0.347–0.020	0.082
	AB	0.033	−0.126–0.192	0.685
	DI	−0.042	−0.195–0.110	0.586

SE, satisfaction with education; AB, academic burnout; DI, dropout intention. All comparisons are based on scalar invariant models. Reference groups were: private university, male, and students who both study and work.

## 4 Discussion

The present study evaluated the psychometric properties and measurement invariance of the *Screening Instrument for Students At-Risk of Dropping Out* in a sample of health sciences students from a public and a private university. Overall, the findings provide evidence supporting the instrument's structural validity, reliability, and cross-group comparability in this context.

First, confirmatory factor analysis supported the original three-factor structure—satisfaction with education, academic burnout, and dropout intention—proposed in the instrument's theoretical framework (20). All factor loadings were statistically significant and of substantial magnitude (*λ* = 0.706–0.964), indicating that the items adequately represent their respective latent constructs. However, examination of inter-factor relationships revealed notable differences compared to the original study. In the present study, a strong and positive association was observed between academic burnout and dropout intention (*r* = 0.776, *p* < .001), consistent with evidence positioning burnout as a proximal predictor of dropout (11, 44). In contrast, satisfaction with education showed a positive but weak association with academic burnout (*r* = 0.240, *p* < .001) and was not significantly associated with dropout intention (*r* = −0.020, *p* = .603).

The absence of a significant association between satisfaction with education and dropout intention observed in this study suggests that, in this context, satisfaction with the educational experience does not necessarily translate into a lower likelihood of dropout. In particular, among health sciences students, the literature has documented the coexistence of positive academic experiences with high levels of demand and emotional exhaustion, associated with academic workload, clinical training, and the structural conditions of academic programs (5). In this regard, it is plausible that students report relatively high levels of satisfaction with their education while simultaneously experiencing academic burnout, which has been consistently identified as a proximal factor of dropout intention (3, 4).

In this regard, the findings do not contradict the initial theoretical structure of the instrument but suggest a reconfiguration in the interaction among constructs, reinforcing the importance of conducting context-specific validations. Although the RMSEA suggested some degree of approximation error, the CFI, TLI, and SRMR values indicated adequate overall model fit. Therefore, the results generally support the adequacy of the proposed three-factor structure and the structural validity of the instrument in this population.

The internal consistency of the instrument was high across all subscales, both in the total sample and in analyses stratified by type of university, with McDonald's omega and ordinal alpha coefficients exceeding recommended thresholds (*ω* = 0.863–0.978; *α* = 0.860–0.978). These findings are consistent with prior evidence reported in the original validation study (20), as well as with recent research in medical students, where adequate levels of internal consistency have been documented for the dimensions of satisfaction with education, academic burnout, and dropout intention (21, 22).

Regarding convergent validity, AVE values exceeded the recommended threshold of 0.50 across all subscales (range = 0.609–0.917), indicating that the latent factors explain a substantial proportion of the variance in their indicators. Complementarily, discriminant validity was supported by HTMT coefficients, which remained below 0.85 across all comparisons, suggesting that the constructs, although related, are not redundant.

This aspect is particularly relevant in the relationship between academic burnout and dropout intention, which showed the strongest associations but remained within acceptable limits of discriminant validity. This pattern is consistent with findings from recent studies (15, 21, 22), which identify academic burnout as a closely related correlate of dropout intention, without complete overlap between the two constructs.

One of the main contributions of this study is the evidence of configural, metric, and scalar invariance across relevant grouping variables, including institutional context, gender, and employment status. The small changes in model fit indices (*Δ*CFI and *Δ*RMSEA) across nested models supported approximate measurement invariance according to the adopted criteria, indicating that imposing equality constraints on factor loadings and item thresholds did not result in substantial deterioration in model fit. Consequently, the instrument demonstrates measurement equivalence under these conditions, allowing for meaningful comparisons of both the construct structure and latent means of satisfaction with education, academic burnout, and dropout intention. This finding supports the use of the instrument for comparative analyses across institutional and demographic subgroups, in line with current methodological recommendations for measurement invariance studies (40).

The establishment of scalar invariance allowed for the examination of latent mean differences, providing information on group differences in the constructs assessed by the instrument. Students from the participating public university reported higher levels of satisfaction with education, academic burnout, and dropout intention compared to those from the participating private university. This pattern suggests that, within the two institutions examined, greater satisfaction with the educational experience coexists with elevated levels of burnout and dropout intention. Previous research has shown that academic burnout is strongly associated with disengagement and dropout-related outcomes, even in contexts where students maintain positive perceptions of their academic experience (15, 22).

Regarding gender differences, the results showed no significant differences in satisfaction with education or academic burnout; however, female students reported lower levels of dropout intention compared to male students. This finding is consistent with previous studies reporting gender differences in academic persistence and dropout-related outcomes (45). These results indicate cross-sectional differences in dropout intention according to gender and should not be interpreted as differences in subsequent dropout behavior.

Interestingly, no significant differences were observed according to employment status. Although balancing academic and work responsibilities has been identified as a factor potentially associated with stress and academic difficulties, its relationship with dropout risk is not uniform and may depend on individual and contextual characteristics (12, 46). Recent studies among health sciences students have shown that academic workload and training conditions may exert a more consistent impact on well-being and performance than other contextual variables, such as employment (47).

Overall, these findings support the proposed multidimensional structure of the instrument and the distinction among satisfaction with education, academic burnout, and dropout intention, in line with theoretical models that emphasize the interaction of psychological, academic, and contextual factors (9, 10). Academic burnout was strongly associated with dropout intention, whereas satisfaction with education was not significantly associated with dropout intention in this sample. These findings should be interpreted as cross-sectional relationships among the assessed constructs rather than as evidence of predictive or protective effects.

From a practical perspective, the validated instrument represents a useful tool for assessing satisfaction with education, academic burnout, and dropout intention among health sciences students. Its demonstrated invariance across different institutions and demographic groups supports its use for comparative assessment of these constructs across student subgroups. Although these dimensions may provide relevant information for understanding processes associated with student persistence, the present findings do not establish the instrument's ability to identify students who will subsequently drop out. Given the importance of student retention in health-related fields, further longitudinal evidence linking instrument scores to subsequent dropout is needed before its use for individual-level screening or targeted intervention decisions can be supported.

Despite its strengths, this study has several limitations. The use of a cross-sectional design precludes causal inferences and limits the ability to examine changes in dropout risk over time. Additionally, the data were based on self-reports, which may be subject to response biases. Measurement invariance was assessed only through the scalar level; although scalar invariance supports latent mean comparisons, strict—or residual— invariance was not examined and could provide additional evidence for comparisons based on observed scores. The academic-program composition differed between institutions, reflecting their distinct health sciences structures. Because programs were not directly comparable across universities, institutional invariance should be interpreted as evidence of instrument performance across two differentiated educational contexts rather than across equivalent academic-program distributions.

The Spanish version underwent expert review and pilot testing for clarity and comprehensibility; however, the adaptation process did not include formal cognitive interviews or quantitative evaluation of cross-language equivalence, which could provide additional evidence regarding its linguistic and cultural equivalence. Finally, consistent with the psychometric scope of the present study, actual dropout, predictive validity, and classification thresholds were not evaluated. Consequently, the present findings provide evidence regarding the measurement properties of the instrument and group comparisons of the assessed constructs, but do not establish its ability to predict or classify subsequent dropout.

Future studies should use longitudinal designs to evaluate the instrument's temporal stability and predictive validity against actual dropout, while incorporating objective academic indicators. Further research should also examine psychological, social, economic, and academic factors associated with student persistence and dropout. Finally, extending validation to other cultural, institutional, and disciplinary contexts across Latin America would strengthen the generalizability of the findings.

## 5 Conclusion

This study provides evidence supporting the structural validity, internal consistency, and measurement invariance of the *Screening Instrument for Students At-Risk of Dropping Out* among health sciences students. The instrument demonstrated a well-supported three-factor structure and approximate measurement invariance across institutional and demographic groups, supporting meaningful comparisons of latent mean levels of satisfaction with education, academic burnout, and dropout intention.

Overall, the findings support the instrument as a psychometrically sound measure of satisfaction with education, academic burnout, and dropout intention and its use for comparative research across relevant student subgroups. However, dropout intention represents a construct related to, but distinct from, actual dropout, and the present cross-sectional study does not establish the instrument's predictive validity for subsequent dropout. Longitudinal studies linking instrument scores with observed dropout are therefore needed before its use for individual-level screening or early identification can be recommended.

## Data Availability

The raw data supporting the conclusions of this article will be made available by the authors, without undue reservation.
