# Supplementary material for: Validity and measurement invariance of the Screening Instrument for Students At-Risk of Dropping Out in health sciences students
Source: Front Med (Lausanne). 2026 Sep 18;13:1941240. doi: 10.3389/fmed.2026.1941240 (PMC13630713; doi:10.3389/fmed.2026.1941240)
Supplement: Supplementary file 1 [file Table1.docx]

Supplementary Material

Supplementary Table S1. Items of the Screening Instrument for Students At-Risk of Dropping Out

| **Item (dimension)** | **English version** | **Spanish version** |
| --- | --- | --- |
| Item 1 (SE) | I am satisfied attending this university. | Estoy satisfecho/a de asistir a esta universidad. |
| Item 2 (SE) | I am satisfied with the education I receive from this university. | Estoy satisfecho/a con la educación que recibo en esta universidad. |
| Item 3 (SE) | My family is pleased with the education I am receiving at this university. | Mi familia está satisfecha con la educación que recibo en esta universidad. |
| Item 4 (SE) | I would recommend this university to a close friend. | Recomendaría esta universidad a un amigo cercano. |
| Item 5 (DI) | I am thinking of changing course. | Estoy pensando en cambiar de carrera. |
| Item 6 (AB) | I am emotionally exhausted studying this course. | Me siento emocionalmente agotado/a estudiando esta carrera. |
| Item 7 (DI) | I have already spoken with friends and/or family about leaving higher education. | Ya he hablado con amigos y/o familiares sobre abandonar la educación superior. |
| Item 8 (AB) | Studying or attending class is stressful for me. | Estudiar o asistir a clases es estresante para mí. |
| Item 9 (AB) | I feel exhausted due to course activities. | Me siento agotado/a debido a las actividades de la carrera. |
| Item 10 (DI) | I feel insecure/indecisive about continuing to study. | Me siento inseguro/a o indeciso/a respecto a continuar estudiando. |
| Item 11 (AB) | I feel stressed every morning about going to university for another day. | Cada mañana me siento estresado/a ante la idea de ir otro día a la universidad. |
| Item 12 (DI) | I am thinking about the possibility of dropping out of higher education. | Estoy pensando en la posibilidad de abandonar la educación superior. |
| *Note.* SE = Satisfaction with education; AB = Academic burnout; DI = Dropout intention. The Spanish column reproduces the version administered to participants, whereas the English column provides its corresponding translation. | | |

Supplementary Table S2. Distribution of participants by academic program

| **Program** | **Private University (n = 437)** | **Public University (n = 320)** |
| --- | --- | --- |
| Medicine | 186 (42.56) | 68 (21.25) |
| Nursing | 177 (40.50) | 41 (12.81) |
| Psychology | 74 (16.93) | — |
| Bacteriology and clinical laboratory | — | 38 (11.88) |
| Physiotherapy | — | 39 (12.19) |
| Speech therapy | — | 39 (12.19) |
| Dentistry | — | 47 (14.69) |
| Occupational therapy | — | 48 (15.00) |
| *Note:* n (%) = frequency and percentage. | | |

Supplementary Table S3. Item-level descriptive statistics of the instrument across the total sample and by university

|  | **Total Sample** | | | **Private University** | | | **Public University** | | |
| --- | --- | --- | --- | --- | --- | --- | --- | --- | --- |
| **Item** | **M (SD)** | **Skewness** | **Kurtosis** | **M (SD)** | **Skewness** | **Kurtosis** | **M (SD)** | **Skewness** | **Kurtosis** |
| Item 1 | 3.88 (1.31) | -1.13 | 0.12 | 3.70 (1.45) | -0.92 | -0.59 | 4.12 (1.04) | -1.29 | 1.18 |
| Item 2 | 3.81 (1.29) | -1.09 | 0.10 | 3.63 (1.44) | -0.86 | -0.66 | 4.05 (1.01) | -1.26 | 1.43 |
| Item 3 | 3.90 (1.31) | -1.18 | 0.22 | 3.72 (1.47) | -0.95 | -0.57 | 4.15 (1.03) | -1.37 | 1.56 |
| Item 4 | 3.82 (1.36) | -1.03 | -0.20 | 3.52 (1.47) | -0.70 | -0.94 | 4.24 (1.05) | -1.53 | 1.76 |
| Item 1 | 1.96 (1.24) | 1.16 | 0.23 | 1.83 (1.18) | 1.37 | 0.79 | 2.14 (1.29) | 0.92 | -0.26 |
| Item 6 | 2.79 (1.33) | 0.12 | -1.15 | 2.61 (1.34) | 0.27 | -1.13 | 3.03 (1.29) | -0.08 | -1.04 |
| Item 7 | 1.91 (1.29) | 1.21 | 0.18 | 1.81 (1.23) | 1.39 | 0.71 | 2.06 (1.36) | 1.01 | -0.34 |
| Item 8 | 2.38 (1.28) | 0.52 | -0.86 | 2.23 (1.24) | 0.65 | -0.73 | 2.57 (1.30) | 0.36 | -0.95 |
| Item 9 | 2.70 (1.32) | 0.12 | -1.19 | 2.56 (1.27) | 0.23 | -1.11 | 2.90 (1.36) | -0.06 | -1.24 |
| Item 10 | 1.92 (1.22) | 1.19 | 0.33 | 1.82 (1.13) | 1.34 | 0.90 | 2.06 (1.31) | 1.00 | -0.27 |
| Item 11 | 2.33 (1.32) | 0.66 | -0.75 | 2.18 (1.25) | 0.81 | -0.43 | 2.53 (1.39) | 0.45 | -1.05 |
| Item 12 | 1.78 (1.17) | 1.51 | 1.28 | 1.69 (1.07) | 1.66 | 1.97 | 1.90 (1.27) | 1.31 | 0.54 |
| *Note:* SE = satisfaction with education; AB = academic burnout; DI = dropout intention. M = Mean; SD = Standard deviation. Skewness and kurtosis describe distributional properties of each item. Values are presented for the total sample (N = 757) and stratified by university (private: n = 437; public: n = 320). All items were measured on a 5-point Likert scale. | | | | | | | | | |

Supplementary Table S4. Standardized factor loadings for the three-factor model

| **Factor** | **Item** | **λ (Standardized)** | ***SE*** | **95% CI** | **p-value** |
| --- | --- | --- | --- | --- | --- |
| **SE** | Item 1 | 0.936 | 0.005 | 0.926–0.947 | < .001 |
|  | Item 2 | 0.964 | 0.004 | 0.957–0.971 | < .001 |
|  | Item 3 | 0.955 | 0.004 | 0.947–0.963 | < .001 |
|  | Item 4 | 0.914 | 0.007 | 0.901–0.927 | < .001 |
| **AB** | Item 6 | 0.706 | 0.020 | 0.666–0.746 | < .001 |
|  | Item 8 | 0.837 | 0.014 | 0.810–0.863 | < .001 |
|  | Item 9 | 0.821 | 0.014 | 0.793–0.850 | < .001 |
|  | Item 11 | 0.844 | 0.013 | 0.818–0.870 | < .001 |
| **DI** | Item 5 | 0.734 | 0.018 | 0.698–0.770 | < .001 |
|  | Item 7 | 0.818 | 0.014 | 0.792–0.845 | < .001 |
|  | Item 10 | 0.915 | 0.008 | 0.899–0.931 | < .001 |
|  | Item 12 | 0.911 | 0.008 | 0.894–0.928 | < .001 |
| *Note:* SE = satisfaction with education; AB = academic burnout; DI = dropout intention. λ = standardized factor loading; *SE* = standard error; 95% CI = 95% confidence interval; p = statistical significance of the factor loadings. | | | | | |
